# Burnout, Job Dissatisfaction, and Mental Health Outcomes Among Medical Students and Health Care Professionals at a Tertiary Care Hospital in Pakistan: Protocol for a Multi-Center Cross-Sectional Study

**DOI:** 10.3389/fpsyg.2019.02552

**Published:** 2019-11-26

**Authors:** Syed Hamza Mufarrih, Aeman Naseer, Nada Qaisar Qureshi, Zohaib Anwar, Nida Zahid, Riaz Hussain Lakdawala, Shahryar Noordin

**Affiliations:** ^1^Department of Biological and Biomedical Sciences, The Aga Khan University Hospital, Karachi, Pakistan; ^2^The Aga Khan University Hospital, Karachi, Pakistan; ^3^Department of Medicine, The Aga Khan University, Karachi, Pakistan; ^4^Department of Surgery, The Aga Khan University Hospital, Karachi, Pakistan

**Keywords:** burnout, depression, stress, anxiety, job satisfaction, health care professionals, physicians, doctors

## Abstract

Burnout, a state of vital exhaustion, has frequently been related to work-related stress and job dissatisfaction. Given the emotionally and physically challenging nature of their work, high rates of burnout have been reported among health care professionals. This may put them at a higher risk for of suffering from adverse mental health outcomes, including depression, anxiety and stress. In our study, we aim to assess the prevalence i of and associations among burnout and job dissatisfaction and adverse mental health outcomes in a developing country, where the challenges faced by the health care system are unique. Facilities are over-burdened and there is a sharp contrast between doctor to patient ratios in developing and developed countries. We plan to conduct a cross sectional study at the largest tertiary care hospital in Pakistan and its peripheral affiliated health centers. A proportionate sampling technique will be employed to include medical and nursing students, interns, residents and consultants. Previously validated questionnaires, including the Maslach Burnout tool, DASS 21, and Job Satisfaction Survey will be disseminated through Survey Monkey. Statistical analysis will be conducted using IBM SPSS Statistics Version 23 to study the association among burnout, job dissatisfaction, adverse health outcomes and demographic and work-related factors This study may begin laying the foundation for prioritizing the novel concept of physician mental health in the developing world. Further research building on to the results of this study will generate evidence to make recommendations about routine screening for mental illness and policy changes in the health care system.

## Introduction

The term “Burnout” was first coined in the 1970s by an American psychologist, Herbertt Freudenberger (InformedHealth.org [Internet], 2006). Since then, over 6000 pieces of literature encompassing the concept of burnout have been published but no unified definition or diagnostic criteria has been established (Schaufeli et al., 2009). The ICD-10 now codes burnout as “a state of vital exhaustion,” affecting both mind and body and includes several elements in common with depression and neglect of physical health (inadequate sleep, lack of exercise, imbalanced diet) (Schaufeli et al., 2009; Korczak et al., 2010; Kaschka et al., 2011; Drummond, 2015; Melnick and Powsner, 2016; Schonfeld and Bianchi, 2016). According to the conservation theory, a state of burnout occurs when an individual’s abilities to cope with physical and emotional stressors are depleted (Hobfoll, 2001; Lesser et al., 2010). While countless physical and emotional stressors may challenge an individual, work-related stress, particularly job dissatisfaction, has often been linked to burnout states (Griffin et al., 2009; Zhou et al., 2017).

Health care provision is a challenging and stressful profession (Ramirez et al., 1996; Familoni, 2008). The serious nature of the work leaves little margin for error (Familoni, 2008). Health care professionals may experience Job dissatisfaction from a failure to cope with competitive work environments, long work hours coupled with overtime and an encroachment on personal life by the psychological burden associated with ethical dilemmas and decision making for patients (Cooper et al., 1989; Theorell et al., 1990; Sutherland and Cooper, 1993; Enzer and Sibbald, 1999; Coomber et al., 2002). Given the emotionally and physically challenging nature of their work, the rates of burnout are alarmingly high in health care professionals, with previous studies reporting rates as high as 54.3% in professionals and 45% in medical students (Bauer et al., 2006; Dyrbye et al., 2014; Lee et al., 2015). In 2019, a meta-analysis on results from 22,778 residents showed that one out of every two residents have suffered from burnout (Low et al., 2019).

Adverse mental health outcomes have been linked to prolonged states of burnout, including depression, anxiety and stress (Prosser et al., 1996; Turnipseed, 1998; Bennett et al., 2005; Peterson et al., 2008). Frequent burnout makes health care professionals more susceptible to a variety of mental illnesses with physical manifestations such as anxiety, depression, insomnia, fatigue, and lethargy (Welp et al., 2015). Depletion of coping abilities may even lead to the development of unhealthy coping strategies, including substance abuse and suicide (Sonneck and Wagner, 1996; Riley, 2004).

Not only do such high levels of stress adversely affect the health and emotional well-being of doctors, they are a direct threat to the quality of care that doctors can provide to their patients While concern for the health of professional caregivers is paramount, such high rates of burnout are a direct threat to the quality of care doctors can provide for their patients (Felton, 1998). Some consequences include early retirements, an increased number of sick leaves from work and reduced daily productivity compromising the delivery of empathy enriched “professional responsibility” by doctors (Lesser et al., 2010; Kumar, 2016). Indeed, higher mortality ratios have been reported in departments with higher burnout rates (Welp et al., 2015).

### Study Aims

While several studies have been conducted to investigate occupational stressors, job satisfaction, burnout and effects on mental health among health care professionals in developed countries (Sonneck and Wagner, 1996; Shapiro et al., 2005; Rössler, 2012), few studies have focused on the developing world where pressures on the health care system are unique. Facilities are over-burdened the doctor to patient ratios are in sharp contrast to the developed world (World Health Organization [WHO], 2013; Khalid and Abbasi, 2018). In 2013, a study conducted in Pakistan reported a rate of 74% among medical and surgical residents (Zubairi and Noordin, 2016).

#### Primary Aim

To study the associations among burnout, job dissatisfaction and mental health outcomes in medical students, interns, residents, fellows and attendings at a tertiary care hospital its peripheral affiliated centers in Pakistan.

#### Secondary Aims

1. To determine the prevalence of job dissatisfaction amongst health care professionals in a tertiary care hospital and its peripheral affiliated centers in Pakistan.
2. To determine the prevalence of burnout amongst health care professionals in a tertiary care hospital and its peripheral branches in Pakistan.
3. Identify the key stressors which are associated with job dissatisfaction amongst health care professionals in a tertiary care hospital and its peripheral branches in Pakistan.

**FIGURE 1:**
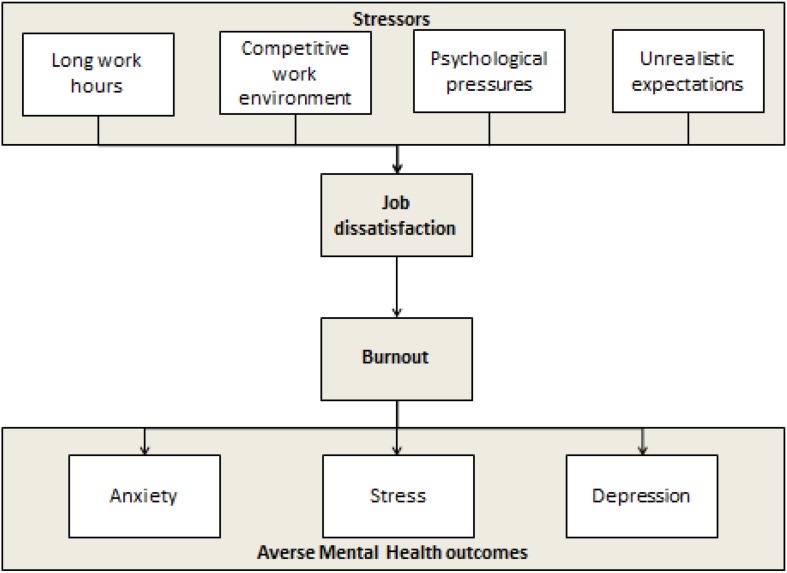
Associations among work-stressors, job dissatisfaction, burnout, and mental health.

Our study attempts to identify the factors which may be associated with burnout, job dissatisfaction and adverse mental outcomes (Figure 1) so that targeted interventions can be made. The results of this study will be beneficial for improving physician and ultimately patient health. The conduction of a study encompassing the topic of physician mental health is among the novel concepts in low middle income countries. If results are favorable, it has potential to scale up to becoming grounds for routine screening and possibly policy changes in the health care system.

## Methods and Analysis

### Study Design

This is a cross sectional study to conducted over a period of 4 months (July 2019–November 2019).

### Study Site

The study will be conducted at the Aga Khan University Hospital and its peripheral affiliated centers including Sultanabad AKU Medical Centre, Metroville Medical Centre and AKU for Women Garden East.

### Participants

#### Inclusion Criteria

1. Students enrolled in the Aga Khan University and medical college (years 1–5).
2. Students enrolled in the Aga Khan school of nursing (years 1–4).
3. Interns employed at Aga Khan University Hospital (1 year contract employees).
4. Residents and attending faculty from Internal Medicine, Emergency medicine, Pediatrics, Surgery, Psychiatry, Neurology, Ophthalmology, Orthopedics, Obstetrics and gynecology, Family medicine and all related specialties employed at AKUH and its peripheral branches.

#### Exclusion Criteria

1. Individuals who do not consent.
2. Students, interns or residents who are on visiting elective rotations at AKUH.

### Sampling Technique and Size

Total population sampling, a subtype of purposive sampling, will be employed. Sample size was calculated using open Epi software version 3.01. Using a level of significance of 5%, precision of 2.5%, design effect of 1, and prevalence of mental health outcomes (depression, anxiety and stress) among students, residents, interns and faculties ranging from 7.9 to 44% (Jadoon et al., 2010; Henning et al., 2014; Washdev et al., 2017; Yahaya et al., 2018), a minimum sample size of 1669 will be needed, with 348 medical students, 348 nursing students, 56 interns, 347 residents, 35 fellows, and 535 faculty members. To account for a response rate less than 100% and limited study duration, questionnaires will be disseminated to all individuals satisfying the eligibility criteria, totaling 2398 health care professionals.

### Data Gathering

#### Procedure

The questionnaires will be disseminated using survey monkey which will utilize electronic consent. The questionnaires are will be provided to the participants in the English Language. There is no need for translation into the local language as all potential participants are fluent in English.

#### Tools

Three questionnaires will be used:

1. *DASS 21* comprises of 21 questions related to symptoms of stress, depression and anxiety. This questionnaire has a sensitivity and specificity of 57 and 76% respectively for depression and 86 and 64% respectively for anxiety (Mitchell et al., 2008). It is estimated to be completed in 6–7 min. The DASS 21 has been used previously in Pakistan, with Chronbach alpha scores of 0.91, 0.84, and 0.90 for depression, anxiety and stress, respectively (Ashiq et al., 2016; Kumar et al., 2019).
2. *Maslach burnout tool* has three sub-scales: EE, DP, and PA. It has a total of 22 questions and will take approximately 6 min to complete. In 2013, Kleijweg et al. (2013) reported the sensitivity and specificity of the MB tool to be 78 and 48% respectively. This tool has frequently been used to assess burnout amongst medical students, doctors and residents in various studies (McManus et al., 2004; Dyrbye et al., 2010). This tool has also been used previously in Pakistan with Chronbach alpha coefficients reported to be 0.75, 0.74, 0.65 for EE, DP and lack of accomplishment, respectively (Abbas et al., 2012; Ali and Ali, 2014; Zubairi and Noordin, 2016).
3. *Job Satisfaction Survey*, developed by PE Spector (1985), has 31 questions and will take approximately 7 min to complete. The test–retest value for this survey is reported to be 0.80–0.64 and the convergent value for contruct validity has been reported to be 0.76 (Koeske et al., 1994). The Cronbach’s alpha for the use of JSS in Pakistan has been reported to be 0.78 (Ali and Ali, 2014; Zubairi and Noordin, 2016).

The JSS and DASS-21 have been used together in several studies (Henning et al., 2014; Sasidharan and Kolasani, 2016).

### Variables, Operational, and Outcome Definitions

#### Independent (Exposure) Variables

##### Demographic

Participants will be requested to fill out a *pro forma* with basic demographic variables including age, gender, marital status, designation, specialty and duration of work/study (Supplementary Appendix 1).

##### Job satisfaction

Job satisfaction is the conglomerate of feelings and beliefs that people have about their current job. A persons’ job satisfaction can range from extreme satisfaction to extreme dissatisfaction (George and Jones, 2008). For assessing the job satisfaction, the sum of the 36-point questionnaire, with each response ranging from 1 to 6, will be calculated and divided into three quartiles (<25, 25–75, >75). All individuals falling in the >75% quartile will be considered as having poor job satisfaction (Supplementary Appendix 2).

##### Burnout

For assessing burnout, we will use the MB tool. Burnout is defined as a state of emotional and physical exhaustion caused by a prolonged period of stress and frustration (Maslach et al., 1996). Responses ranged between 0 and 3 describe severity of burnout. MB tool scores output in three dimensions – EE, DP, and PA, which will be transformed into dummy categorical variables using the cutoff values for doctors, as recommended by Maslach et al. (1986).

#### Dependent (Outcome) Variables

##### Depression

Depression is an illness that is marked by feelings of sadness, worthlessness, or hopelessness, as well as with problems concentrating and remembering details. For the assessment of depression and anxiety, we will use the questionnaire, DASS-21by Henry (Henry and Crawford, 2005). The DASS-21 is a 4 point questionnaire with severity scores ranging from (InformedHealth.org [Internet], 2006; Schaufeli et al., 2009; Melnick and Powsner, 2016; Schonfeld and Bianchi, 2016) and severity is rated using the sum of responses to the 21 questions (Table 1 and Supplementary Appendices 3, 4).

**TABLE 1:** DASS severity rating (multiply summed score by × 2) (Henry and Crawford, 2005).

**Severity**	**Depression**	**Anxiety**	**Stress**
Normal	0–9	0–7	0–14
Mild	10–13	8–9	15–18
Moderate	14–20	10–14	19–25
Severe	21–27	15–19	26–33
Extremely severe	28+	20+	34+

##### Anxiety

Anxiety is a feeling of fear or apprehension about what is to come (Wurman et al., 2001). A score greater than 20 in the DASS-21 will be interpreted as anxiety.

##### Stress

A score of greater than 34 in the DASS-21 will be interpreted as stress.

### Data Management

Only the primary investigator and data analyst will have access to the electronic data which will be kept in a password protected database.

### Statistical Analysis Plan

This is an observation, cross-sectional study to estimate the prevalence of burnout, job dissatisfaction and adverse health outcomes amongst health care professionals and assess associations among them and with other potential demographic factors.

Analysis will be performed using IBM SPSS Statistics version 23. Descriptive statistics will be applied to categorical variables as frequencies or proportions and as measures of central tendency to quantitative variables [mean ± SD or Median (IQR) as appropriate]. Mean scores will be reported for depression, anxiety and stress. One way ANOVA/Kruskil Wallis test will be used to compare differences amongst scores of medical students, interns, residents and faculty different groups. In order to assess the relationship between depression, anxiety and stress correlation analysis will also be performed using Pearson or Spearman rank correlation coefficients as appropriate. Univariate and MLR will be performed to evaluate the effect of independent variables (age, gender, marital status and designation) on the outcomes. Adjusted β-coefficients with their 95% CI will be reported. A *p*-value of <0.05 will be considered statistically significant. The results of this study will be depicted in the dummy tables and graphs as shown in Supplementary Appendix 5.

### Caveats and Potential Pitfalls

The study is limited to a single city and affiliated hospitals of a single institute and may not be a reflection of the entire Pakistani population. For this reason, this study must be replicated in smaller cities across several hospitals to improve generalizability. Furthermore, due to time constrains of health care workers, filling in a questionnaire with over 50 questions may limit response rates. In response rates are too low to meet the minimum sample size, the designated study duration can be extended and frequent reminders can be sent to participants through Survey Monkey. Lastly, our study is a pilot study which will only be studying associations among burnout, job dissatisfaction and adverse mental outcomes. To establish a stronger statistical relationships, long term cohort studies with adequate control of confounders need to be conducted.

### Ethics and Dissemination

The Ethical approval for this study has been obtained from the institutional review board of the Aga Khan University (ERC number: 2019-1126-3077). Electronic consent form will be obtained from each participant through Survey Monkey prior to the study. A unique study identification number will be assigned to each participant. Permissions from the authors of the DASS-21 questionnaire and the JSS questionnaire have been taken to conduct our study using their questionnaires. MBI-Burnout tool will be purchased from the Consulting Psychologists Press^1^.

### Implications for Patients

It will be a cross sectional study design where participants will answer questionnaires regarding job satisfaction, burnout and mental health. The participants will be health care professionals and there will be no direct involvement of patients. Long term, however, addressing physician burnout may improve quality of health care provided to patients. The study findings will be disseminated through the university newsletter and mental health conferences following its publication in a national or international journal. Participants who wish to see their results will receive their report via email.

## Data Availability Statement

The raw data supporting the conclusions of this manuscript will be made available by the authors, without undue reservation, to any qualified researcher.

## Ethics Statement

The studies involving human participants were reviewed and approved by Institutional Review Board, The Aga Khan University Hospital, Karachi (ERC: 2019-1126-3077). The patients/participants provided their written informed consent to participate in this study.

## Author Contributions

SM: study design and protocol writing. AN: protocol writing. NQ: study design and protocol review. ZA: study design. NZ: protocol review. RL and SN: protocol review and supervisor. All authors contributed to manuscript revision, and read and approved the submitted version.

## Conflict of Interest

The authors declare that the research was conducted in the absence of any commercial or financial relationships that could be construed as a potential conflict of interest.

## Footnotes

1 http://www.mindgarden.com/products/mbi.htm

## Abbreviations

AKUH: Aga Khan University Hospital
DASS-21: depression anxiety stress scale 21
DP: depersonalization
EE: emotional exhaustion
ERC: ethical review committee
IQR: inter quartile range
JSS: job satisfaction survey
MB tool: Maslach burnout tool
MLR: multiple linear regression
PA: personal accomplishment.
