# Supplementary material for: Burnout, Job Dissatisfaction, and Mental Health Outcomes Among Medical Students and Health Care Professionals at a Tertiary Care Hospital in Pakistan: Protocol for a Multi-Center Cross-Sectional Study
Source: Front Psychol. 2019 Nov 26;10:2552. doi: 10.3389/fpsyg.2019.02552 (PMC6888812; doi:10.3389/fpsyg.2019.02552)
Supplement: Supplementary file 1 [file Data_Sheet_1.docx]

**Demographics questionnaire**

**Date: Time: Area:**

**Study title:** Burnout, job dissatisfaction and its relationship with adverse mental health outcomes among health care professionals in a Tertiary care Hospital, Karachi, Pakistan: A cross-sectional study.

**Age:** _____ Gender (circle one): **M F**

**Marital status: _________**

**Designation: Student, Resident, House officer, Faculty, Other _________________**

**Specialty: _______________

Year of residency/ medical school: ________________

Number of years working in the health care profession: _______________**

**Appendix 1:** Demographics of the sample population.
