# Supplementary material for: Burnout, Job Dissatisfaction, and Mental Health Outcomes Among Medical Students and Health Care Professionals at a Tertiary Care Hospital in Pakistan: Protocol for a Multi-Center Cross-Sectional Study
Source: Front Psychol. 2019 Nov 26;10:2552. doi: 10.3389/fpsyg.2019.02552 (PMC6888812; doi:10.3389/fpsyg.2019.02552)
Supplement: Supplementary file 2 [file Data_Sheet_2.doc]

|  | **JOB SATISFACTION SURVEY**  Paul E. Spector  Department of Psychology  University of South Florida  Copyright Paul E. Spector 1994, All rights reserved. |  |
| --- | --- | --- |
|  | PLEASE CIRCLE THE ONE NUMBER FOR EACH QUESTION THAT COMES CLOSEST TO REFLECTING YOUR OPINION  ABOUT IT. | Disagree very much  Disagree moderately  Disagree slightly  Agree slightly  Agree moderately  Agree very much |
| 1 | I feel I am being paid a fair amount for the work I do. | 1 2 3 4 5 6 |
| 2 | There is really too little chance for promotion on my job. | 1 2 3 4 5 6 |
| 3 | My supervisor is quite competent in doing his/her job. | 1 2 3 4 5 6 |
| 4 | I am not satisfied with the benefits I receive. | 1 2 3 4 5 6 |
| 5 | When I do a good job, I receive the recognition for it that I should receive. | 1 2 3 4 5 6 |
| 6 | Many of our rules and procedures make doing a good job difficult. | 1 2 3 4 5 6 |
| 7 | I like the people I work with. | 1 2 3 4 5 6 |
| 8 | I sometimes feel my job is meaningless. | 1 2 3 4 5 6 |
| 9 | Communications seem good within this organization. | 1 2 3 4 5 6 |
| 10 | Raises are too few and far between. | 1 2 3 4 5 6 |
| 11 | Those who do well on the job stand a fair chance of being promoted. | 1 2 3 4 5 6 |
| 12 | My supervisor is unfair to me. | 1 2 3 4 5 6 |
| 13 | The benefits we receive are as good as most other organizations offer. | 1 2 3 4 5 6 |
| 14 | I do not feel that the work I do is appreciated. | 1 2 3 4 5 6 |
| 15 | My efforts to do a good job are seldom blocked by red tape. | 1 2 3 4 5 6 |
| 16 | I find I have to work harder at my job because of the incompetence of people I work with. | 1 2 3 4 5 6 |
| 17 | I like doing the things I do at work. | 1 2 3 4 5 6 |
| 18 | The goals of this organization are not clear to me. | 1 2 3 4 5 6 |

|  | PLEASE CIRCLE THE ONE NUMBER FOR EACH QUESTION THAT COMES CLOSEST TO REFLECTING YOUR OPINION  ABOUT IT.  Copyright Paul E. Spector 1994, All rights reserved. | Disagree very mcuh  Disagree moderately  Disagree slightly  Agree slightly  Agree moderately  Agree very much |
| --- | --- | --- |
| 19 | I feel unappreciated by the organization when I think about what they pay me. | 1 2 3 4 5 6 |
| 20 | People get ahead as fast here as they do in other places. | 1 2 3 4 5 6 |
| 21 | My supervisor shows too little interest in the feelings of subordinates. | 1 2 3 4 5 6 |
| 22 | The benefit package we have is equitable. | 1 2 3 4 5 6 |
| 23 | There are few rewards for those who work here. | 1 2 3 4 5 6 |
| 24 | I have too much to do at work. | 1 2 3 4 5 6 |
| 25 | I enjoy my coworkers. | 1 2 3 4 5 6 |
| 26 | I often feel that I do not know what is going on with the organization. | 1 2 3 4 5 6 |
| 27 | I feel a sense of pride in doing my job. | 1 2 3 4 5 6 |
| 28 | I feel satisfied with my chances for salary increases. | 1 2 3 4 5 6 |
| 29 | There are benefits we do not have which we should have. | 1 2 3 4 5 6 |
| 30 | I like my supervisor. | 1 2 3 4 5 6 |
| 31 | I have too much paperwork. | 1 2 3 4 5 6 |
| 32 | I don't feel my efforts are rewarded the way they should be. | 1 2 3 4 5 6 |
| 33 | I am satisfied with my chances for promotion. | 1 2 3 4 5 6 |
| 34 | There is too much bickering and fighting at work. | 1 2 3 4 5 6 |
| 35 | My job is enjoyable. | 1 2 3 4 5 6 |
| 36 | Work assignments are not fully explained. | 1 2 3 4 5 6 |

**Appendix 2:** Job Satisfaction Survey by PE Spector (1)


**1) Spector, P. E. (1985). Measurement of human service staff satisfaction: 644 development of the job satisfaction survey. Am. J. Commun. Psychol. 13,693–713. doi: 10.1007/bf00929796**
