# Supplementary material for: Burnout, Job Dissatisfaction, and Mental Health Outcomes Among Medical Students and Health Care Professionals at a Tertiary Care Hospital in Pakistan: Protocol for a Multi-Center Cross-Sectional Study
Source: Front Psychol. 2019 Nov 26;10:2552. doi: 10.3389/fpsyg.2019.02552 (PMC6888812; doi:10.3389/fpsyg.2019.02552)
Supplement: Supplementary file 3 [file Data_Sheet_3.pdf]

DASS21

Name: *Date:*

Please read each statement and circle a number 0, 1, 2 or 3 which indicates how much the statement applied to you *over the past week*. There are no right or wrong answers. Do not spend too much time on any statement.

The rating scale is as follows:

0 Did not apply to me at all
1 Applied to me to some degree, or some of the time
2 Applied to me to a considerable degree, or a good part of time
3 Applied to me very much, or most of the time

1	I found it hard to wind down	0	1	2	3
2	I was aware of dryness of my mouth	0	1	2	3
3	I couldn't seem to experience any positive feeling at all	0	1	2	3
4	I experienced breathing difficulty (eg, excessively rapid breathing, breathlessness in the absence of physical exertion)	0	1	2	3
5	I found it difficult to work up the initiative to do things	0	1	2	3
6	I tended to over-react to situations	0	1	2	3
7	I experienced trembling (eg, in the hands)	0	1	2	3
8	I felt that I was using a lot of nervous energy	0	1	2	3
9	I was worried about situations in which I might panic and make a fool of myself	0	1	2	3
10	I felt that I had nothing to look forward to	0	1	2	3
11	I found myself getting agitated	0	1	2	3
12	I found it difficult to relax	0	1	2	3
13	I felt down-hearted and blue	0	1	2	3
14	I was intolerant of anything that kept me from getting on with what I was doing	0	1	2	3
15	I felt I was close to panic	0	1	2	3
16	I was unable to become enthusiastic about anything	0	1	2	3
17	I felt I wasn't worth much as a person	0	1	2	3
18	I felt that I was rather touchy	0	1	2	3
19	I was aware of the action of my heart in the absence of physical exertion (eg, sense of heart rate increase, heart missing a beat)	0	1	2	3
20	I felt scared without any good reason	0	1	2	3
21	I felt that life was meaningless	0	1	2	3

Appendix 3: DASS-21 survey by Mitchell et. al. (1)

1) Mitchell, M., Burns, N., and Dorstyn, D. (2008). Screening for depression and anxiety in spinal cord injury with DASS-21. Spinal Cord. 46, 547–551. doi: 10.1038/sj.sc.3102154
