# Supplementary material for: Burnout, Job Dissatisfaction, and Mental Health Outcomes Among Medical Students and Health Care Professionals at a Tertiary Care Hospital in Pakistan: Protocol for a Multi-Center Cross-Sectional Study
Source: Front Psychol. 2019 Nov 26;10:2552. doi: 10.3389/fpsyg.2019.02552 (PMC6888812; doi:10.3389/fpsyg.2019.02552)
Supplement: Supplementary file 4 [file Data_Sheet_4.pdf]

A Guide to the Depression, Anxiety and Stress Scale (DASS 21)

Introduction

The DASS 21 is a 21 item self report questionnaire designed to measure the severity of a range of symptoms common to both Depression and Anxiety.

In completing the DASS, the individual is required to indicate the presence of a symptom over the previous week. Each item is scored from 0 (did not apply to me at all over the last week) to 3 (applied to me very much or most of the time over the past week).

The essential function of the DASS is to assess the severity of the core symptoms of Depression, Anxiety and Stress. Accordingly, the DASS allows not only a way to measure the severity of a patient's symptoms but a means by which a patient's response to treatment can also be measured.

The DASS and Diagnosis

Although the DASS may contribute to the diagnosis of Anxiety or Depression, it is not designed as a diagnostic tool. Indeed, a number of symptoms typical of Depression such as sleep, appetite and sexual disturbances, are not covered by the DASS and will need to be assessed independently. The DASS is not meant to replace a comprehensive clinical interview.

Suicide

Suicidality is not assessed by the DASS. Accordingly, the clinician will need to address directly this important symptom of Depression in their clinical interview.

How often to use the DASS?

Although the DASS can provide a comparison of symptoms from week to week, it is best given on first presentation and again after a period of time has lapsed long enough for the chosen treatment to have effect. In the case of anti depressant medication, the second administration should be between the 2-4 week period after the individual has commenced taking the medication. This period is long enough for most anti depressants to be expected to show some change in the patient.

Scoring the DASS

A plastic scoring template is provided to simplify scoring the scale. To use the plastic template, place it over the completed response form so that the borders are aligned. The scale to which each item belongs is listed to the left of the rating scales, by the letters D (Depression), A (Anxiety) and S (Stress). For each scale (D, A & S) sum the scores for identified items.

Because the DASS 21 is a short form version of the DASS (the Long Form has 42 items), the **final score of each item groups (Depression, Anxiety and Stress) needs to be multiplied by two (x2).**

Interpreting the DASS

Once multiplied by 2, each score can now be transferred to the DASS profile sheet, enabling comparisons to be made between the three scales and also giving percentile rankings and severity labels.

DASS Severity Ratings

(Don't forget to multiply summed scores by x 2)

Severity	Depression	Anxiety	Stress
Normal	0-9	0-7	0-14
Mild	10-13	8-9	15-18
Moderate	14-20	10-14	19-25
Severe	21-27	15-19	26-33
Extremely Severe	28+	20+	34+

As previously mentioned, the DASS should not be used on its own to assess the presence or absence of Depression or Anxiety. High scores on the DASS would certainly alert the clinician to a high level of distress in the patient and this would need to be explored further within the interview process. Similarly, low scores on the DASS should not be a substitute for a comprehensive clinical interview.

High DASS scores which are not changing, may prompt the clinician to look for explanations and perhaps augment dosages or change medication. Here again, the DASS should be interpreted along side the clinical interview.

Changes in scores in one scale (EG: Depression), with consistently high and unchanging scores in another scale (Anxiety) may alert the clinician to pay particular attention to the presence of a co-existing anxiety disorder which may need specific treatment in its own right.

Similarly, decreasing Depression scores along side unchanging Stress scores may alert the clinician to the presence of some life event or problem, which may need to be addressed directly.

Fernando Gomez
Consultant Clinical Psychologist

Appendix 4: DASS-21 scoring key
