# Supplementary material for: Burnout, Job Dissatisfaction, and Mental Health Outcomes Among Medical Students and Health Care Professionals at a Tertiary Care Hospital in Pakistan: Protocol for a Multi-Center Cross-Sectional Study
Source: Front Psychol. 2019 Nov 26;10:2552. doi: 10.3389/fpsyg.2019.02552 (PMC6888812; doi:10.3389/fpsyg.2019.02552)
Supplement: Supplementary file 5 [file Data_Sheet_5.docx]

**APPENDIX 5: Dummy tables of results**

|  | **Students** | | **Residents** | | **Attendings** | **Total** |
| --- | --- | --- | --- | --- | --- | --- |
|  | Medical students | Nursing students | Interns | Residents |  |  |
| **N**  **Response Rate** |  |  |  |  |  |  |
| **Age** <25 26-35  36-45  45-55  >55 |  |  |  |  |  |  |
| **Marital status** Single Married Separated Widowed Children at home |  |  |  |  |  |  |
| **Time since beginning of job/ school** <2 years 3-4 years 5-10 years  11-20 years  >20 years |  |  |  |  |  |  |
| **Percent of time spent at work/ school per week (make it work hours per week- that’s standard)** 1-25% 26-50% 51-75% 76-100% |  |  |  |  |  |  |
| **Job satisfaction (Mean score)** | N/A | N/A |  |  |  |  |
| **Emotional Exhaustions** |  |  |  |  |  |  |
| **Depersonalization** |  |  |  |  |  |  |
| **Personal Accomplishment** |  |  |  |  |  |  |
| **Anxiety + % (n)** |  |  |  |  |  |  |
| **Stress + %(n)** |  |  |  |  |  |  |
| **Depression + %(n)** |  |  |  |  |  |  |
| **Table 1: Characteristics of the populations** | | | | | |  |

|  | Medical students | | | | | | Nursing Students | | | | | |
| --- | --- | --- | --- | --- | --- | --- | --- | --- | --- | --- | --- | --- |
| **Subscale** | High scores | | Moderate scores | | Low scores | | High scores | | Moderate scores | | Low scores | |
|  | n (%) | Mean + SD | n (%) | Mean + SD | n (%) | Mean + SD | n (%) | Mean + SD | n (%) | Mean + SD | n (%) | Mean + SD |
| EE |  |  |  |  |  |  |  |  |  |  |  |  |
| DP |  |  |  |  |  |  |  |  |  |  |  |  |
| PE |  |  |  |  |  |  |  |  |  |  |  |  |
| **Table 2: Comparison of burnout among medical students and nursing students** EE: Emotional Exhaustion; DP: Depression; PE: Personal accomplishment | | | | | | | | | | | | |

|  | Residents + Interns | | | | | | Consultants | | | | | |
| --- | --- | --- | --- | --- | --- | --- | --- | --- | --- | --- | --- | --- |
| **Subscale** | High scores | | Moderate scores | | Low scores | | High scores | | Moderate scores | | Low scores | |
|  | n (%) | Mean + SD | n (%) | Mean + SD | n (%) | Mean + SD | n (%) | Mean + SD | n (%) | Mean + SD | n (%) | Mean + SD |
| EE |  |  |  |  |  |  |  |  |  |  |  |  |
| DP |  |  |  |  |  |  |  |  |  |  |  |  |
| PE |  |  |  |  |  |  |  |  |  |  |  |  |
| **Table 3: Comparison of burnout among residents + interns and consultants** EE: Emotional Exhaustion; DP: Depression; PE: Personal accomplishment | | | | | | | | | | | | |

|  | Medical students | | | | | | Nursing Students | | | | | |
| --- | --- | --- | --- | --- | --- | --- | --- | --- | --- | --- | --- | --- |
| **Subscale** | Anxiety | | Depression | | Stress | | Anxiety | | Depression | | Stress | |
|  | n | (%) | n | (%) | n | (%) | n | (%) | n (%) | (%) | n | (%) |
| Normal |  |  |  |  |  |  |  |  |  |  |  |  |
| Mild |  |  |  |  |  |  |  |  |  |  |  |  |
| Moderate |  |  |  |  |  |  |  |  |  |  |  |  |
| Severe |  |  |  |  |  |  |  |  |  |  |  |  |
| Extremely Severe |  |  |  |  |  |  |  |  |  |  |  |  |
| **Table 4: Comparison of adverse health outcomes among medical students and nursing students** | | | | | | | | | | | | |

|  | Residents | | | | | | Consultants | | | | | |
| --- | --- | --- | --- | --- | --- | --- | --- | --- | --- | --- | --- | --- |
| **Subscale** | Anxiety | | Depression | | Stress | | Anxiety | | Depression | | Stress | |
|  | n | (%) | n | (%) | n | (%) | n | (%) | n (%) | (%) | n | (%) |
| Normal |  |  |  |  |  |  |  |  |  |  |  |  |
| Mild |  |  |  |  |  |  |  |  |  |  |  |  |
| Moderate |  |  |  |  |  |  |  |  |  |  |  |  |
| Severe |  |  |  |  |  |  |  |  |  |  |  |  |
| Extremely Severe |  |  |  |  |  |  |  |  |  |  |  |  |
| **Table 5: Comparison of adverse health outcomes among residents and consultants** | | | | | | | | | | | | |

|  | **Job satisfaction** | | | | | |
| --- | --- | --- | --- | --- | --- | --- |
|  | **1^st^ quartile  High job satisfaction** | | **2^nd^ & 3^rd^ quartile  Moderate job satisfaction** | | **4^th^ quartile  Poor Job satisfaction** | |
|  | n | (%) | n | (%) | n | (%) |
| **Specialty** | | | | | | |
| **Surgery** |  |  |  |  |  |  |
| **Medicine** |  |  |  |  |  |  |
| **Family medicine** |  |  |  |  |  |  |
| **Psychiatry** |  |  |  |  |  |  |
| **Pediatrics** |  |  |  |  |  |  |
| **Obstetrics and Gynecology** |  |  |  |  |  |  |
| **Neurology** |  |  |  |  |  |  |
| **Category** | | | | | | |
| **Residents** |  |  |  |  |  |  |
| **Consultants** |  |  |  |  |  |  |
| **Interns** |  |  |  |  |  |  |
| **Off-campus Secondary care** |  |  |  |  |  |  |
| **Table 5: Comparison of job dissatisfaction among residents and nursing consultants 1^st^ quartile =** (0-25%); 2^nd^ & 3^rd^ quartile = (26-75%); 4^th^ quartile = (76-100%) | | | | | | |

| Category | **High scores (BO)** | | **High scores  (BO + JDS)** | | **High scores  (BO + AHO)** | | **High scores (JDS + AHO)** | | **High scores  (BO + JDS + AHO)** | |
| --- | --- | --- | --- | --- | --- | --- | --- | --- | --- | --- |
|  | n (%) | p-value | n (%) | p-value | n (%) | p-value | n (%) | p-value | n (%) | p-value |
| **Medical students** |  |  | N/A | N/A |  |  | N/A | N/A | N/A | N/A |
| **Nursing students** |  |  | N/A | N/A |  |  | N/A | N/A | N/A | N/A |
| **Interns** |  |  |  |  |  |  |  |  |  |  |
| **Residents** |  |  |  |  |  |  |  |  |  |  |
| **Attending’s** |  |  |  |  |  |  |  |  |  |  |
| **Table 6:** Association of burnout, job dissatisfaction and adverse health outcomes | | | | | | | | | | |

**Figure 1:** Scatter diagram of association of high burnout rates with adverse health outcomes.
X-axis: Burnout; Y-axis: Adverse health outcomes **(These are template diagrams and do not represent any data)**

**Figure 2:** Scatter diagram of association of job dissatisfaction rates with adverse health outcomes.
X-axis: Job-dissatisfaction; Y-axis: Adverse health outcomes **(These are template diagrams and do not represent any data)**
